# Angiographic evaluation of traumatic arterial injuries of the upper limbs: a retrospective study

**DOI:** 10.34172/jcvtr.2020.11

**Published:** 2019-10-28

**Authors:** Abolhassan Shakeri-Bavil, Sina Zarrintan

**Affiliations:** ^1^Section of Angiography, Imam Hospital, Tabriz University of Medical Sciences, Tabriz, Iran; ^2^Department of General & Vascular Surgery, Shahid Beheshti University of Medical Sciences, Tehran, Iran

**Keywords:** Upper Limb, Trauma, Angiography, Orthopedic, Artery

## Abstract

***Introduction:*** The aim of this study was to evaluate the mechanism and type of upper limb arterial trauma in Iranian population.

*** Methods:*** Fifty-one patients with upper limb trauma were evaluated over a 4-year period with conventional angiography at the Tabriz Imam Hospital, Iran.

*** Results:*** Twenty-four patients (19 men, 5 women with a mean age of 27.5 ± 11.8 years) had arterial injuries. Blunt trauma was more frequent than penetrating trauma (87.5%). The most cause of trauma was traffic accidents and the brachial artery was the most frequently affected artery. In 87.5% cases associated bone injuries were observed.

*** Conclusion:*** Patients with blunt upper limb injuries must be evaluated for vascular integrity timely, especially in traffic accidents because vascular injuries after traffic accidents need to be referred to vascular centers. The results of this article are of potential use and clinical importance because precise diagnosis of vascular insults are essential to restore injured extremities.

## Introduction

Trauma and especially vascular injuries are public health problems worldwide. The incidence of vascular injuries in the civilian population has increased steadily over the past decades due to an increase in automobile accidents and criminal violence and its frequency is high especially in summers.^1,2^ It is approximately 1%-4% after traffic accidents in peace times.^3^ Nearly 90% of arterial injuries affect the limbs. Lower limbs are usually injured in military events and upper limb injuries, mainly in civilian event.^4^ Acute arterial injuries of the upper limbs include nearly half of the civilian arterial injuries in the United States.^5^

Vascular injuries of upper limbs are associated with remarkable mortality and morbidity. Mangled extremities with vascular injury increase amputation rate up to 40%.^1^ But recent significant developments in vascular surgery have reduced the rate of limb loss to less than 10-15% in civilian arterial injuries.^5^ However, in some countries trauma resulting from road traffic crashes is still the most common indication of major limb amputation.^6^

But the prognosis and the outcome of traumatic vascular injuries depend on the cause of injury, and an appropriate knowledge of vascular injury mechanisms helps surgeons applying proper diagnostic procedures and related therapeutic plans. There are remarkable differences in management and therapeutic methods of arterial injuries according to the mechanism of injuries, and mechanism of injury seems to vary in different parts of the world.^5,7,8^ Since little is known about the incidence or nature of arterial limb injuries in Iran, the aim of this study was to evaluate the mechanism and type of upper limb arterial trauma in northwest Iran.

## Patients and Methods

This study was conducted in Tabriz Imam Khomeini hospital, the largest tertiary level referral center for trauma in northwest Iran. During the 4-year period between October 2006 and September 2009, 51 patients with upper limb vascular trauma were evaluated with conventional angiography. The patients were either first assessed by a vascular surgeon or were referred from an orthopedic surgeon after absent distal pulses were noted in a patient with a fracture or fracture/dislocation of the upper limb. When vascular injuries were suspected, angiography was considered as the first step. In all patients with associated bone injury, reduction of joint dislocation and immobilization by external fixation always preceded vascular repair.

The age and gender of patients as well as the injured artery and mechanism of injury were recorded. All angiograms were performed via the femoral artery and a multipurpose catheter was positioned in the origin, proximal or portion of subclavian artery. Nonionic radiographic contrast media was injected.

Statistical analysis of the results was performed by using SPSS 13.0. *P* values less than 0.05 were considered statistically significant.

## Results

Twenty-four of the patients (47%) had arterial injuries. They consisted of 19 men and 5 women with a mean age of 27.5 ± 11.8 years, ranging from 2 to 50 years. The average age of the men was 27.0 ± 11.9 and for the women was 29.4 ± 12.4 and there was no significant statistical difference between them. The mechanism of trauma was blunt in 87.5% of cases and the majority of them were related to motor vehicle accidents (62.5%) (Table 1). Women were afflicted exclusively following car accidents.

**Table 1:** Characteristics of the arterial injuries of the upper limbs

**Characteristic**	**No. (%)**
Mechanism of injury	
Motorcycle accident	8 (33.3)
Car accident	7 (29.2)
Falling	3 (12.5)
Industrial accident	3 (12.5)
Iatrogenic	1 (4.0)
Gunshot	1 (4.0)
Self-injury	1 (4.0)
Anatomical sites of arterial injuries	
Brachial	16 (66.7)
Subclavian	6 (25.0)
Axillary	1 (4.2)
Ulnar	1 (4.2)
Associated orthopedic injuries	
Fx. Supracondylar of humerus	5 (20.8)
Fx. Middle third of humerus	2 (8.3)
Fx. Clavicle	2 (8.3)
Fx. Radius and olecranon	1 (4.2)
Fx. Mid shaft of humerus	1 (4.2)
Fx. Distal third of humerus	3 (12.5)
Fx. Proximal third of humerus	1 (4.2)
Fx. Ulna and radius	1 (4.2)
Fx. Clavicle and rib	3(12.5)
Fx. Rib	1(4.2)
Posterior dislocation of elbow	2 (8.3)
Types of arterial injuries	
Distal occlusion	9 (37.5)
Middle occlusion	4 (16.7)
Proximal occlusion	1 (4.2)
Occlusion of the origin	3 (12.5)
Active bleeding	1 (4.2)
Spasm and compression	2 (8.3)
Complete occlusion	4 (16.7)

Fx, fracture.

**Figure 1:**
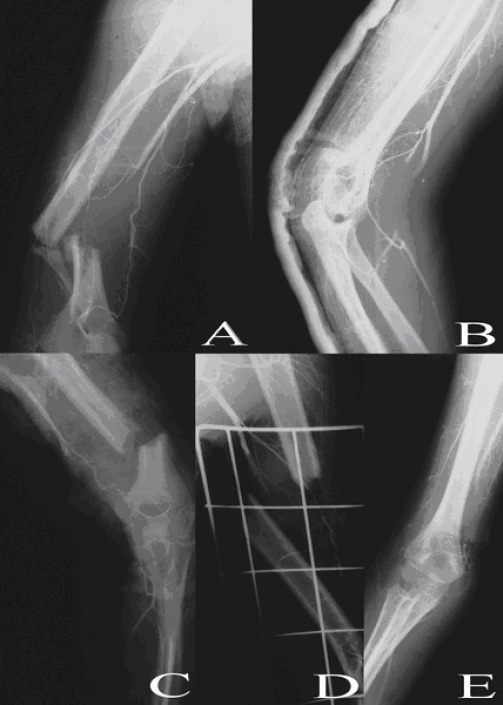
Angiograms of occluded brachial arteries; Occlusion of the middle third of the brachial artery together with the fracture of the distal shaft of the humerus (A); Occlusion of the distal third of the brachial artery together with the supracondylar humeral fracture after reduction (B); occlusion of the distal third of the brachial artery together with the fracture of the distal third of the humerus (C); occlusion of the proximal third of the brachial artery together with the fracture of the shaft of the humerus (D); occlusion of the distal third of the brachial artery together with the reduced dislocation of the elbow (E).

**Figure 2:**
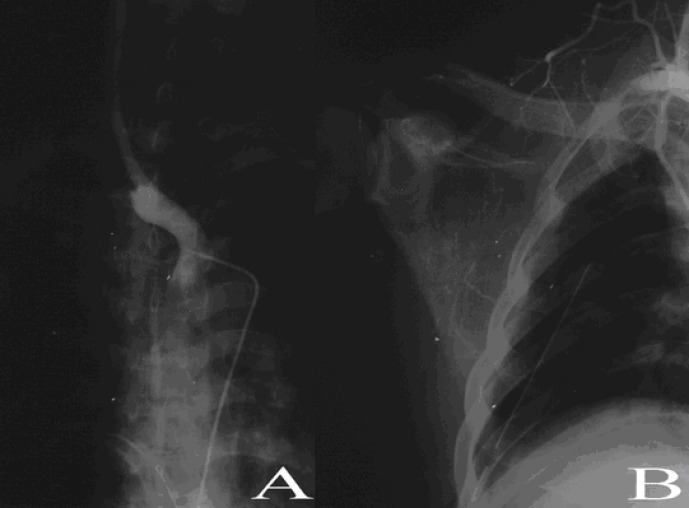
Angiogram of occluded subclavian arteries; Occlusion of right subclavian artery after the branching of vertebral artery (A); occlusion of right subclavian artery 1 cm after the branching of vertebral artery (B).

**Figure 3:**
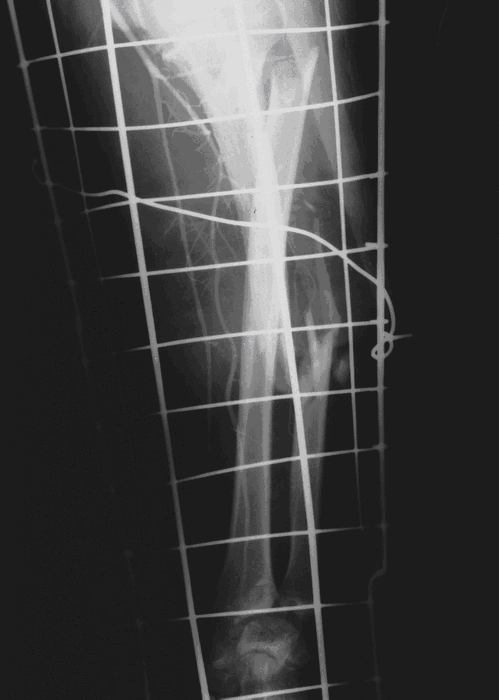
Angiogram of occluded ulnar artery in the middle third of the forearm. Note the fractures of the radius and ulna.

The brachial artery was the most frequently affected artery (66%) followed by subclavian artery (25%). Figures 1 and 2 show sample angiograms of brachial and subclavian arteries respectively. Figure 3 also shows a sample angiogram of the ulnar artery.

The most common affected arteries were brachial and subclavian with equal proportions in females. The anatomical sites of the injuries are shown in Table 1.

The associated bone injuries were fractures (79%) and dislocations (8.5%).

In 12.5% of patients neither fracture nor dislocation was observed. Various types of arterial injuries are also listed in Table 1. There was no significant relationship between the type of arterial injuries and associated bone injuries.

## Discussion

The upper extremity is more endanger during a vehicle crash which a concern knows the position of arm on a vehicle door/window, as an increasing factor for injury.^1^ In our study, similar to other studies,^7-10^ young men were affected most frequently with traumatic upper limb arterial injuries. The most common mechanism was blunt trauma and penetrating trauma was less common which in pediatrics the rate of penetrating trauma is high.^11^

Penetrating trauma was the predominant mechanism in reports from the Unites States (70-90%)^12-14^ and it was also the same in a study of 868 patients from Ireland^10^ Two studies from Turkey^7,9^ found the frequency of penetrating trauma to be 83% and 80% respectively. In a study in Saudi Arabia^8^ penetrating trauma was the cause of injuries in 95% of cases. However, in some countries blunt trauma has been reported as the most common cause of injury. In a Canadian study^15^ blunt trauma was the most common mechanism of upper limb arterial injuries (76%). In a study performed in England,^16^ blunt trauma was the cause of injury in 73% of cases, and in Thailand,^17^ in 57% of all injuries. In the majority, all of these studies specify that penetrating trauma was due to gunshot or stab wounds and blunt trauma was the result of traffic accidents or falls.

As seen, all the mechanisms of vascular injuries show a regional variation and differ remarkably in different countries dependent on cultures and laws. For example, in the Unites States where carrying a gun is allowed, the incidence of gunshot injuries is higher than traffic accidents. Conversely in some countries, especially in Iran, the rate of traffic accidents is higher and therefore it is the primary cause of limb injury.^2,18^ In rural India where the incidence of high-speed automobile accidents and civilian violence is low, falling from a height is the most common cause of injury.^19^

In this study traumatic injuries equally affected left and right limbs in males and females, and all injuries in female patients were due to road traffic accidents. In a study in Saudi Arabia, most of the vascular injuries in the female patients were due to household trauma and the mechanism of trauma was significantly sex-dependent.^8^ In the same study, the right upper arm was the most frequently affected side in females (83%). These findings may due to the fact that women are not allowed to drive in Saudi Arabia and the right upper limb which is more involved in household activities was the most frequently affected side in women.

In our study the brachial artery was the most often injured artery (67%) followed by the subclavian artery (25%) which is consistent with the majority of other studies in this field.^7-10,15^

Associated fracture or dislocation in our study was common (87.5%), while in other studies it varies from 12.5% to 70%. This difference, we believe, can be attributed to the mechanism of injury. In blunt trauma, especially due to road accidents, associated bone injuries increase. In studies in which blunt trauma was the predominant cause, the rate of fracture or dislocation was higher. When penetrating trauma (e.g., gunshot or stabbing) was the main cause, the probability of associated bone injuries was less.

As expected, the mechanism of trauma has therapeutic implications, in blunt trauma the rate of ischemia increases while in penetrating trauma hemorrhage is more frequent. Associated bone injuries occur commonly in blunt trauma. Thus, arterial injuries could be missed in blunt trauma, therefore the risk of ischemia increases. In conclusion, patients with blunt upper limb injuries must be evaluated for vascular integrity timely, especially in traffic accidents and be referred to vascular centers if needed.^20^

## Competing interests

None.

## Ethical approval

The protocol of this study was approved by Tabriz University of Medical Sciences, Tabriz, Iran.
